# On the identity of Blanco’s *Cissus frutescens* and its correct name in *Melicope* (Rutaceae) with neotypification of *Cissus arborea* Blanco

**DOI:** 10.3897/phytokeys.58.5847

**Published:** 2016-01-12

**Authors:** Marc S. Appelhans, Jun Wen

**Affiliations:** 1 Department of Systematic Botany, Albrecht-von-Haller Institute of Plant Sciences, University of Göttingen, Untere Karspüle 2, 37073 Göttingen, Germany; 2 Department of Botany, Smithsonian Institution, PO Box 37012, Washington, DC 20013-7012, USA

**Keywords:** Blanco, Cissus, Melicope confusa, Melicope frutescens, Nomenclature, Philippines, Rutaceae, Vitaceae

## Abstract

The names *Cissus frutescens* and *Cissus arborea* have a long history of confusion. *Cissus frutescens* Blanco belongs to the genus *Melicope* (Rutaceae) and we herein correct a nomenclatural mistake made by T.G. Hartley in the revision of *Melicope*. The name *Melicope confusa* (Merr.) P.S. Liu was accepted for this taxon by Hartley. However, *Cissus frutescens* Blanco represents the earliest name for this entity and a new combination, *Melicope frutescens* (Blanco) Appelhans & J.Wen is herein proposed. Neotypification of *Cissus arborea* Blanco is also provided.

## Introduction

The name *Cissus frutescens* Blanco was published in 1837 in the first edition of Francisco Manuel Blanco’s Flora de Filipinas (Blanco 1837). The description in Spanish was relatively short and did not cite any collection. Blanco’s second edition of this work was published in 1845 shortly after his death and included several name changes without comments or reference to the first edition (Merrill 1905). Among these names is *Cissus frutescens*, which was changed to *Cissus arborea* Blanco, but the treatment of the taxon remained identical to that in the first edition (Blanco 1845).

After Blanco’s death, an addendum to the Flora de Filipinas was written by Fernandez-Villar, who considered *Cissus frutescens*/*Cissus arborea* conspecific with *Evodia roxburghiana* (Cham.) Benth. [*Evodia* =*Euodia*^1^, Rutaceae] (Fernandez-Villar, 1877-1883). *Euodia roxburghiana* is currently known as *Melicope lunu-ankenda* (Gaertn.) T.G.Hartley, and interestingly, only a single specimen of this widespread species was cited from the Philippines in the latest revision of *Melicope* and *Euodia* (Hartley, 2001), suggesting that it might be rare in the Philippines. This was highlighted by Merrill, who stated that the species was “not definitely known from the Philippines” (Merrill 1918: 198).

**Figure 1.**
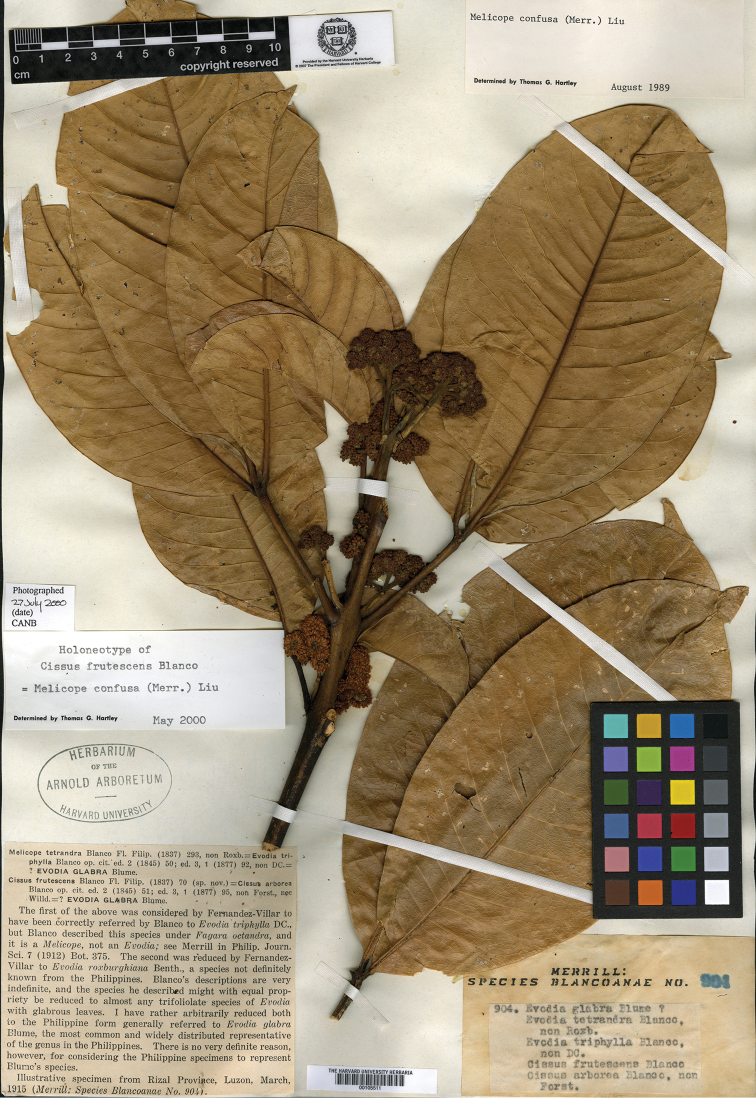
The neotype of *Melicope frutescens* (Blanco) Appelhans & J.Wen (A).

In 1918 Merrill also treated *Cissus frutescens*/*Cissus arborea* as conspecific with *Euodia glabra* Blume, noting that “Blanco’s descriptions were very indefinite, and the species Blanco described might with equal propriety be reduced to almost any trifoliolate species of *Evodia* with glabrous leaves” (Merrill 1918: 198). In 1918, Merrill mentioned a collection of *Cissus frutescens* that he collected in the vicinity of Blanco’s locality for this species (*Merrill, Species Blancoanae No. 904*, Fig. 1). Merrill considered this collection an “illustrative specimen” with duplicates deposited at A, GH, K, L, NSW, NY, P, US and W. In 1922, Merrill published the new species *Euodia confusa* Merr. where *Merrill 904* was listed along with many other specimens. Liu (1962) transferred *Euodia confusa* to *Melicope*, a decision which is in agreement with recent revisionary (Hartley and Stone 1989; Hartley 2001) and molecular phylogenetic studies (Appelhans et al. 2014a, b) where a total of seven species, restricted to New Guinea, northern Australia and several Pacific island groups are recognized in *Euodia*. All species of *Euodia* from the Philippines have been transferred to *Melicope* (Hartley and Stone 1989; Hartley 2001).

The latest revision of *Melicope* (Hartley 2001) included the lectotypification and the neotypification of the names *Melicope confusa* and *Cissus frutescens*, respectively. Hartley (2001) reported that the type of *Euodia confusa* (*Ramos 15055*, PNH) was lost and therefore he chose *Borden 3045* (NY) among the paratypes, as its lectotype. He also designated *Merrill 904* as the neotype of *Cissus frutescens*, which he placed as a synonym of *Melicope confusa* (Hartley 2001). *Merrill 904* is the “illustrative specimen” that Merrill provided for Blanco’s names and which was listed in the protologue of *Euodia confusa* (Merrill 1922).

Until the neotypification of *Cissus frutescens* (Hartley 2001), the status of Blanco’s names was unclear. Blanco’s species descriptions were not detailed enough to differentiate among several species of *Euodia*/*Melicope*, and his collections were lost. However, by assigning a neotype to *Cissus frutescens*, Hartley (2001) definitely associated the specimen *Merrill 904* to this taxon name. By the principle of nomenclatural priority the species epithet *frutescens* must be used for the entity of *Melicope confusa*, as *Melicope confusa* represents a synonym of *Cissus frutescens*. The epithet *frutescens* is not pre-empted in *Melicope*.

Three other authors used the name *Cissus arborea*. *Cissus arborea* Forssk. (Forsskål 1775) is a synonym of *Salvadora persica* L. (Salvadoraceae; Roemer and Schultes 1818); *Cissus arborea* Willd. ex Roem. & Schult. is a synonym of *Ardisia guianensis* (Aubl.) Mez. (Mez 1901); and *Cissus arborea* (L.) Des Moul. is a synonym of *Nekemias arborea* (L.) J.Wen & Boggan (Wen et al. 2014), so the Des Moulins’ taxon remains the only *Cissus arborea* that actually represents a Vitaceae species.

## Taxonomic treatment

### Melicope frutescens

Taxon classificationPlantaeSapindalesRutaceae

(Blanco) Appelhans & J.Wen comb. nov.

urn:lsid:ipni.org:names:77151881-1

#### Basionym.

*Cissus frutescens* Blanco, Flora de Filipinas, ed. 1: 70. 1837. **Type**: Philippines. Luzon: Rizal, Mar 1915, *Merrill: Species Blancoanae No. 904* (neotype: A!, designated by Hartley 2001, p. 220; isoneotypes: GH!, K, L, NSW, NY!, P!, US!, W).

*Cissus arborea* Blanco, nom. illeg., Flora de Filipinas, ed. 2: 51. 1845 (non Forssk., 1775). **Type**: Philippines. Luzon: Rizal, Mar 1915, *Merrill: Species Blancoanae No. 904* (neotype: A!, designated here; isoneotypes: GH!, K, L, NSW, NY!, P!, US!, W).

*Euodia confusa* Merr., Philipp. J. Sci. 20: 391. 1922. *Melicope confusa* (Merr.) P.S. Liu, III. Native Introd. Lign. Pl. Taiwan 2: 876. 1962. **Type**: Philippines. Luzon: Bataan, *Borden FB 3045* (lectotype: NY!, designated by Hartley, 2001, p. 220; isolectotypes: BO, SING, US!).

In addition to its distribution in the Philippines, *Melicope frutescens* is known to occur in Borneo, Sulawesi and the Moluccas. It typically grows in the lowlands but reached elevations of up to 1800 m in the Philippines. The species occurs in primary, secondary, and disturbed rainforests.

## Supplementary Material

XML Treatment for Melicope frutescens

## Footnotes

1 Fernandez-Villar used the old spelling *Evodia*, whereas the conserved spelling is *Euodia*

## References

[B1] Appelhans MS Wen J Wagner WL (2014a) A molecular phylogeny of *Acronychia*, *Euodia*, *Melicope* and relatives (Rutaceae) reveals polyphyletic genera and key innovations for species richness. Molecular Phylogenetics and Evolution 79: 54–68. doi: 10.1016/j.ympev.2014.06.014 24971739 10.1016/j.ympev.2014.06.014

[B2] Appelhans MS Wen J Wood KR Allan GJ Zimmer EA Wagner WL (2014b) Molecular phylogenetic analysis of Hawaiian Rutaceae (*Melicope*, *Platydesma* and *Zanthoxylum*) and their different colonisation patterns. Botanical Journal of the Linnean Society 174: 425–448. doi: 10.1111/boj.12123

[B3] Blanco FM (1837) Flora de Filipinas: segun el sistema sexual de Linneo, ed. 1 Imprenta de Sto. Thomas, 887 pp.

[B4] Blanco FM (1845) Flora de Filipinas: segun el sistema sexual de Linneo, ed. 2 D. Miguel Sanchez, 619 pp.

[B5] Forsskål P (1775) Flora Aegyptiaco-Arabica. Ex Officina Mölleri, Hauniæ [Copenhagen], 1–32. doi: 10.5962/bhl.title.41

[B6] Fernandez-Villar C (1877-1883) Novissima appendix ad floram Philippinarum R. P. Fr. Emmanuelis Blanco. Plana, Manila, 375 pp.

[B7] Hartley TG Stone BC (1989) Reduction of *Pelea* with new combinations in *Melicope* (Rutaceae). Taxon 38: 119–123. doi: 10.2307/1220910

[B8] Hartley TG (2001) On the taxonomy and biogeography of *Euodia* and *Melicope* (Rutaceae). Allertonia 8: 1–328.

[B9] Liu PS (1962) Illustrations of Native and Introduced Ligneous Plants of Taiwan, vol. 2. College of Agriculture, National Taiwan University, Taipei, Taiwan, 686 pp.

[B10] Merrill ED (1905) A review of the identifications of the species described in Blanco’s Flora de Filipinas. Bureau of Public Printing, Manila, 132 pp. doi: 10.5962/bhl.title.58554

[B11] Merrill ED (1918) Species Blancoanae. A critical revision of the Philippine species of plants described by Blanco and by Llanos. Bureau of Public Printing, Manila, 423 pp. doi: 10.5962/bhl.title.2116

[B12] Merrill ED (1922) New or noteworthy Philippine plants XVII. Philippine Journal of Science 20: 367–476.

[B13] Mez C (1901) IX. Myrsinaceae. In: Urban I (Ed.) Symbolae Antillanae seu fundamenta Florae Indinae Occidentalis, Vol. 2 Borntraeger, Lipsiae [Leipzig], 389–433.

[B14] Roemer JJ Schultes JA (1818) Caroli a Linné. Systema vegetabilium: secundum classes, ordines, genera, species. Cum characteribus, differentiis et synonymiis, vol. 3 Sumtibus J.G. Cottae, Stuttgart, 888 pp.

[B15] Wen J Boggan J Nie Z-L (2014) Synopsis of *Nekemias* Raf., a segregate genus from *Ampelopsis* Michx. (Vitaceae) disjunct between eastern/southeastern Asia and eastern North America, with ten new combinations. PhytoKeys 42: 11–19. doi: 10.3897/phytokeys.42.7704 25383008 10.3897/phytokeys.42.7704 PMC4223361

